# Household food security and urinary phthalate metabolite concentrations among US adults: Evidence from NHANES 2003–2018

**DOI:** 10.1097/EE9.0000000000000512

**Published:** 2026-09-01

**Authors:** Marshae Nickelberry, Emma V. Preston, Marlee R. Quinn, Kimberly L. Parra, Izzuddin M. Aris, Ayishat Yussuf, Michele R. Hacker, Emily Oken, Paige L. Williams, Eric B. Rimm, Thomas F. McElrath, Tamarra James-Todd

**Affiliations:** a Department of Environmental Health, Harvard T.H. Chan School of Public Health, Boston, Massachusetts; b Division of Chronic Disease Research Across the Lifecourse, Department of Population Medicine, Harvard Medical School and Harvard Pilgrim Health Care Institute, Boston, Massachusetts; c Spelman College, Atlanta, Georgia; d Department of Obstetrics and Gynecology, Beth Israel Deaconess Medical Center, Boston, Massachusetts; e Department of Obstetrics, Gynecology and Reproductive Biology, Harvard Medical School, Boston, Massachusetts; f Department of Epidemiology, Harvard T.H. Chan School of Public Health, Boston, Massachusetts; g Department of Biostatistics, Harvard T.H. Chan School of Public Health, Boston, Massachusetts; h Department of Nutrition, Harvard T.H. Chan School of Public Health, Boston, Massachusetts; i Division of Maternal-Fetal Medicine, Department of Obstetrics and Gynecology, Brigham and Women’s Hospital, Boston, Massachusetts

**Keywords:** Phthalates, Food insecurity, biomarkers, NHANES, Environmental exposure, Socioeconomic status, Health disparities

## Abstract

**Background:**

Phthalates are ubiquitous environmental contaminants and established endocrine disruptors with disproportionate exposure among disadvantaged populations. Food insecurity, a marker of socioeconomic deprivation, may be a modifiable contributor to disparate phthalate exposure among adults. Still, associations between food insecurity and phthalate concentrations remain unclear.

**Methods:**

We analyzed cross-sectional data from 13,024 adults in eight cycles of the National Health and Nutrition Examination Survey (2003–2018). Using multivariable linear regression models, we assessed associations between food security status (four categories) and percent difference in urinary phthalate metabolite concentrations (spot sample), adjusting for sociodemographic factors, survey year, and urinary creatinine. We assessed effect modification by sex and race/ethnicity.

**Results:**

Compared with adults with high food security (70.5%), those experiencing very low food security (7.1%) had significantly higher urinary concentrations of monobenzyl phthalate (MBzP: 25.1%; 95% confidence interval [CI]: 13.2%, 38.2%), mono-n-butyl phthalate (MnBP: 9.6%; 95% CI: 1.1%, 18.8%), higher ∑di-2-ethylhexyl phthalate (12.1%; 95% CI: 2.4%, 22.7%), ∑Butyls (11.4%; 95% CI: 3.9%, 19.4%), and ∑Plastic-associated metabolites (16.0%; 95% CI: 6.4%, 26.4%). Sex and race/ethnicity, respectively, modified the food security–metabolite associations. Stronger associations were observed among females (MBzP, ∑di-2-ethylhexyl phthalate, ∑Butyls, and ∑Plastics), as well as non-Hispanic White, non-Hispanic Black, and other race individuals (MnBP and MBzP).

**Conclusion:**

Lower food security was associated with elevated concentrations of several phthalate biomarkers among US adults, with stronger associations seen in certain population subgroups. These findings highlight the need to further investigate food insecurity as a modifiable risk factor to decrease phthalate exposure disparities.

**What this study adds:**

Previous studies have linked correlates of food security, such as ultra-processed and fast-food intake to urinary phthalate metabolites; endocrine disruptors associated with adverse health outcomes. To our knowledge, no study has evaluated household food security using validated measures as a predictor of phthalate biomarker levels. Using 2003–2018 National Health and Nutrition Examination Survey data from 13,024 adults, we found reduced food security was associated with higher concentrations of several phthalate biomarkers. Stronger inverse associations were seen for food security and phthalate metabolites in females and certain race/ethnicity subgroups. Our study highlights a novel and modifiable social determinant of phthalate exposure.

## Introduction

Food security is challenging in many populations, with lower food security being associated with adverse health outcomes.^1–3^ The extent to which these associations might be mediated by greater exposure to harmful environmental chemicals^4^ linked to poor diet remains uncertain. Approximately 18 million households across the United States had reduced food security or food insecurity, an increase from the previous year and pre-coronavirus disease 2019 (COVID-19) pandemic levels.^3^ The distribution of individuals experiencing food insecurity across the US is not homogenous, with racial, ethnic, and socioeconomic disparities persisting. Non-Hispanic Black and Hispanic individuals, as well as individuals experiencing poverty, are more likely to experience food insecurity.^5^ Additionally, households with unmarried female heads and children are also more likely to experience food insecurity,^6^ as opposed to unmarried male heads of households or married couples with children.^6,7^

Individuals experiencing food insecurity may alternate between a state of hunger and periods of consumption of low-cost, calorie-dense, nutrient-poor foods to supplant hunger.^2,8^ This phenomenon, often referred to as the “substitution effect,” results in a shift away from consumption of higher quality, less calorically dense foods such as produce and certain sources of protein to more energy-dense foods, often higher in highly processed carbohydrates, saturated fats, and sodium.^2^ This shift often reflects key characteristics individual’s experiencing food insecurity face that restrain food choices: limited financial resources and reduced structural access to retailers (i.e., supermarkets) that provide healthy food options^9^ and potential increased access to retailers with more readily available processed options.^9^

Along with the potential nutritional deficits associated with altered dietary intake experienced with this substitution effect,^10^ the increased consumption of processed foods may also result in greater uptake of environmental endocrine-disrupting chemicals used in food processing and packaging, as highly processed foods often are contaminated from food contact materials through processing and food packaging.^4^ Among those chemicals are higher molecular weight phthalates (di-2-ethylhexyl phthalate [DEHP]),^11^ commonly used as plasticizers to provide flexibility to polyvinyl chloride materials used for shelf-stable packaging or fast food wrappers.^12–15^ Lower molecular weight phthalate compounds, including butylbenzyl phthalate (BBzP), may also be used in food contact materials.^16^

Phthalates can easily leach into food sources during the processing stages or from food packaging^17,18^ and, upon uptake, are readily metabolized and excreted in urine within hours following exposure.^19^ While exposure from co-occurring sources (including personal care product use and housing materials)^20–22^ may occur, the dietary pathway is particularly relevant in the context of food insecurity, given the established shift toward processed and packaged foods among food-insecure households. While previous studies have found associations between processed food intake and higher phthalate metabolite concentrations,^14,15,23^ to our knowledge, no study has investigated food security explicitly as an upstream socioeconomic determinant in association with phthalate exposure.

Despite ubiquitous exposure to phthalates, sex and race/ethnicity are also important to consider in the context of phthalate exposure disparities. Females have higher urinary phthalate metabolite concentrations than males,^24^ potentially reflecting greater exposure through personal care products and, less studied, through dietary sources linked to food security disparities. Racial and ethnic disparities in phthalate exposure have similarly been documented, with non-Hispanic Black and Hispanic individuals exhibiting higher concentrations relative to non-Hispanic White individuals,^20^ patterns that also closely mirror those observed for food security and socioeconomic position overall. The extent to which these disparities may modify the role of food security on phthalate exposure is unclear.

In this cross-sectional study, we investigated the extent to which household food security status is associated with urinary phthalate metabolite concentrations among adult participants in the NHANES study from 2003 to 2018. We hypothesized that lower levels of food security (i.e., reduced food security) would be associated with higher food-associated urinary phthalate metabolite concentrations (e.g., ∑*DEHP*). We also hypothesized that sex and race/ethnicity would modify these associations, with higher levels seen among females, as well as non-Hispanic Black and Hispanic populations, as the dose–response relationship among subgroups may differ at higher levels of food insecurity.

## Methods

### Study design

This study utilized cross-sectional 2003–2018 cycle data from NHANES, a US-based study designed to assess the health and nutritional status of participants via interviews and physical examinations.^21^ Interviews included detailed collection of demographic, socioeconomic, dietary, and health-related questions,^25^ while physical examinations consisted of medical, dental, and physiological measurements, as well as laboratory tests.^26,27^ The Centers for Disease Control and Prevention (CDC) has published detailed protocols.^27^

Participants were eligible for inclusion in the present study if they were 18 years of age or older (n = 17,003), had urinary creatinine, food security, and covariate data (n = 14,920), urinary phthalate metabolite data (n = 13,192), and were not outliers (n = 13,024) (Supplemental Figure1, https://links.lww.com/EE/A450). Informed consent for study participants was obtained in writing as per NHANES protocol, and the National Center for Health Statistics Research Ethics Review Board approved the study protocol.^28^

### Food security

NHANES assessed food security using the US Household Food Security Survey Module (FSSM),^29^ a validated questionnaire ranging from 10 to 18 items depending on the status of children in the household, developed by the US Department of Agriculture (USDA).^29^ The FSSM measures the presence of household food security during the past 12 months, and responses represent all household members, not just NHANES participants.^29^ The USDA derived a categorical variable with four levels from the FSSM responses to reflect household food security: household high food security (reference), household marginal food security, household low food security, and household very low food security.^30^ For conciseness, we will refer to these levels as high, marginal, low, and very low food security, respectively, throughout the remainder of this paper.

### Urinary phthalate metabolite concentrations

NHANES measured phthalate metabolite (ng/mL) concentrations in spot urine samples using solid-phase extraction coupled with high-performance liquid chromatography electrospray ionization tandem mass spectrometry (HPLC–ESI–MS/MS)^21^ at the CDC’s National Center for Environmental Health (Atlanta, GA).^31^ Phthalates are rapidly metabolized and excreted in urine; therefore, urinary phthalate metabolites, which reflect exposure to the parent compounds and are thought to represent the biologically active forms, were measured to minimize exposure misclassification.^21,32^ For the present analysis, we selected metabolites that were consistent across cycle years and restricted to those that were detected in >60% of sample concentrations. As a result, we restricted our analysis to eight urinary phthalate metabolites: mono-ethyl phthalate (MEP), mono-n-butyl phthalate (MnBP), mono-isobutyl phthalate (MiBP), mono-benzyl phthalate (MBzP), mono-2-ethylhexyl phthalate (MEHP), mono-(2-ethyl-5-hydroxyhexyl) phthalate (MEHHP), mono-(2-ethyl-5-oxohexyl) phthalate (MEOHP), and mono-(3-carboxypropyl) phthalate (MCPP). Metabolite concentrations below the lower limit of detection (LOD) were replaced with LOD/√2.^33^ We removed metabolite concentrations above five standard deviations of the mean.^21^

These eight urinary phthalate metabolites were assessed as individual metabolites or as molar sums of metabolites representing singular pathways or highly correlated groups. The di-2-ethylhexyl phthalate pathway was defined as: (∑DEHP: MEHP/278.34 + MEHHP/294.34 + MEOHP/292.33 + MECPP/308.33).^34^ Butyl phthalate metabolites were calculated as (∑Butyls: MnBP/222.24 + MiBP/222.24 + MBzP/256.25).^35^ Source-specific measures were also created for personal care product-associated metabolites (∑PCP= MnBP/222.24 + MEP/194.18 + MiBP/222.24),^36^ as well as plastics-associated metabolites (∑Plastics = MEHP/278.34 + MEHHP/294.34 + MEOHP/292.33 + MECPP/308.33 + MBzP/256.25).^37^

### Covariates

We obtained covariates from computer-assisted surveys administered by NHANES personnel.^38^ We used directed acyclic graphs based on previous literature^12–15,25^ to identify potential confounders. Specifically, we considered age, sex, education, race, ethnicity, and family income–to-poverty (FIP) ratio as potential confounders. NHANES calculates an FIP ratio based on the US Department of Health and Human Services poverty guidelines.^39^ We also created dummy variables for the eight cycle years. Finally, we adjusted for urinary creatinine (ng/mL) as a precision covariate to adjust for urinary dilution, in line with previous analyses.^40,41^

### Statistical analysis

We summarized population characteristics across food security levels by calculating frequencies (percentages) or means (standard deviations) for sociodemographic factors and other covariates. Urinary phthalate metabolite concentrations were natural log transformed, and we reported detection frequencies (%>LOD), and 25th, geometric mean (GM), and 75th percentiles. Analyte correlations were assessed with Spearman coefficients. To estimate differences in mean urinary phthalate metabolite levels across food security categories (marginal, low, very low) versus high food security (reference), we fit multivariable linear regression models for each individual log-transformed urinary phthalate metabolite and summary measures. We reported exponentiated estimated coefficients as the percent (%) difference: 100 × (exp(*β*) − 1). Positive values indicate higher concentrations in the comparison group; negative values indicate lower.

In addition to the creatinine-adjusted models, we also constructed fully adjusted models with most covariates modeled according to their NHANES categorization: sex (female (ref), male), education level (< high school diploma (ref), high school diploma, some college, ≥ college diploma), a composite variable of race/ethnicity (non-Hispanic White (ref), non-Hispanic Black, Mexican-American, other Hispanic, Other), and FIP ratio (<1 (ref), 1–1.99, 2–2.99, 3–3.99, 4–4.99, and ≥5.0).^38^ We categorized age into equally spaced quantiles based on the analytic sample’s distribution (18–30 (ref), 31–46, 47–63, 64–85). We also created dummy variables for the survey cycle years (2003–2004, 2005–2006, 2007–2008, 2009–2010, 2011–2012, 2013–2014, 2015–2016, 2017–2018) to account for previously demonstrated temporal trends and fluctuations. Finally, we adjusted for urinary creatinine (ng/mL) in minimally adjusted and fully adjusted models. We calculated variance inflation factors and generalized variance inflation factors for all fully adjusted models. All values fell between 1.1 and 1.5, well below the threshold of concern (variance inflation factor < 5), and all models converged without issues, indicating that collinearity did not adversely impact the analysis.

To assess potential effect measure modification by sex, race, and ethnicity, we first modeled food security as a categorical variable and included interaction terms to estimate stratum-specific marginal means. These categorical estimates are useful for visualizing the data, but small cell sizes in some strata may lead to wide confidence intervals and imprecise estimates. Thus, global tests of categorical interaction may be underpowered or yield spurious findings in sparse strata. Our primary formal inference, therefore, relies on modeling food security as an ordinal variable and testing an ordinal × five-level interaction. This approach leverages the natural ordering of food security, reduces the number of parameters, and stabilizes estimates. This ordinal approach also assumes equal spacing between categories but provides the necessary statistical power to detect consistent trends across modifiers of interest. We considered a conservative *P* value < 0.10 for statistical significance to account for potential limitations in power.

To address the observed increase in certain low-molecular-weight phthalate metabolites, which may be used as medication coatings, we conducted post hoc analyses of supplement use, OC use, and fasting status as predictors of urinary phthalate metabolite concentrations. We used R Studio 4.3.2 for analyses. All analyses utilized the R “survey” package and NHANES multistage probability weights to account for the complex multistage sampling design and to yield results that are representative of the US population aged 18 years and older.^13^

## Results

### Study population

Among 13,024 study participants, 51.4% were female, and 82% were below age 64 (Table 1). Individuals aged 18–30 were most likely to report low (34.5%) or very low food security (35.5%). Decreased food security was also disproportionately prevalent in lower-income groups: 45.7 of those with very low food security had an FIP ratio below 1.0, as opposed to 8.8% in the high food security group. Similarly, racial and ethnic disparities were seen. The proportion of non-Hispanic White individuals decreased from 73.9% in the high food security group to 52.6% in the very low food security group. In contrast, the proportions of non-Hispanic Black (9.4%–18.1%), Mexican American (5.8%–13.3%), and other Hispanic (3.7%–10.0%) individuals increased with decreasing food security (Table 1).

**Table 1.** Demographic characteristics (%) by food security status, NHANES (2003–2018)

	Overall	High (Ref)	Marginal	Low	Very Low
	(N = 13,024)	N = 9,058	N = 1,460	N = 1,589	N = 917
	N (%)	N (%)	N (%)	N (%)	N (%)
Sex					
Female	6,662 (51.4%)	4,566 (50.9%)	776 (53.3%)	843 (53.2%)	477 (52.0%)
Male	6,362 (48.6%)	4,492 (49.1%)	684 (46.7%)	746 (46.8%)	440 (48.0%)
Age (years)					
18–30	3,429 (25.9%)	2,108 (23.0%)	504 (37.0%)	521 (34.5%)	296 (35.5%)
31–46	3,366 (29.6%)	2,237 (28.8%)	407 (33.5%)	462 (32.7%)	260 (29.8%)
47–63	3,218 (26.5%)	2,261 (27.6%)	304 (18.1%)	401 (24.4%)	252 (27.4%)
64–85	3,011 (18.0%)	2,452 (20.6%)	245 (11.4%)	205 (8.4%)	109 (7.3%)
Race/ethnicity					
Non-Hispanic White	5,601 (67.9%)	4,413 (73.9%)	429 (48.5%)	426 (44.5%)	333 (52.6%)
Non-Hispanic Black	2,873 (11.5%)	1,837 (9.4%)	385 (17.8%)	407 (18.8%)	244 (18.1%)
Mexican-American	2,092 (8.2%)	1,168 (5.8%)	338 (15.4%)	415 (18.7%)	171 (13.3%)
Other Hispanic	1,076 (5.1%)	606 (3.7%)	158 (9.9%)	201 (10.0%)	111 (10.0%)
Other incl. multiracial	1,382 (7.3%)	1,034 (7.2%)	150 (8.4%)	140 (8.1%)	58 (6.0%)
Highest educational attainment					
Less than high school	3,301 (16.2%)	1,790 (12.2%)	506 (26.3%)	654 (33.0%)	351 (30.3%)
High school graduate or equivalent	3,358 (25.2%)	2,253 (23.6%)	417 (30.9%)	435 (29.2%)	253 (31.6%)
Some college	3,590 (30.3%)	2,537 (30.2%)	400 (31.8%)	382 (28.5%)	271 (32.5%)
College degree or more	2,775 (28.3%)	2,478 (34.0%)	137 (11.0%)	118 (9.2%)	42 (5.6%)
Family income: ratio					
0–0.99	2,910 (14.8%)	1,260 (8.8%)	511 (28.4%)	670 (35.3%)	469 (45.7%)
1–1.99	3,496 (20.8%)	2,025 (16.2%)	520 (33.4%)	611 (38.0%)	340 (38.5%)
2–2.99	1,904 (14.6%)	1,426 (14.6%)	231 (18.9%)	181 (14.1%)	66 (7.2%)
3–4.99	2,452 (24.0%)	2,148 (27.7%)	156 (14.4%)	113 (11.4%)	35 (7.2%)
5.0 or greater	2,262 (25.8%)	2,199 (32.8%)	42 (4.9%)	14 (1.1%)	7 (1.5%)
Cycle year					
2003–2004	1,505 (11.4%)	1,163 (12.2%)	111 (7.4%)	153 (9.1%)	78 (10.2%)
2005–2006	1,508 (12.0%)	1,148 (13.0%)	149 (9.7%)	143 (8.3%)	68 (7.0%)
2007–2008	1,721 (12.2%)	1,291 (13.1%)	173 (10.6%)	166 (9.2%)	91 (8.1%)
2009–2010	1,801 (12.2%)	1,227 (12.2%)	195 (11.8%)	232 (12.7%)	147 (11.4%)
2011–2012	1,614 (12.9%)	1,104 (12.7%)	195 (14.6%)	189 (11.4%)	126 (14.7%)
2013–2014	1,750 (13.2%)	1,197 (12.7%)	199 (14.8%)	217 (14.9%)	137 (14.3%)
2015–2016	1,579 (12.8%)	964 (11.7%)	214 (15.3%)	257 (16.8%)	144 (16.4%)
2017–2018	1,546 (13.5%)	964 (12.4%)	224 (15.9%)	232 (17.6%)	126 (18.0%)

Count (weighted frequency) and proportion (weighted percentage) per food security category reported in each cell.

Detection frequencies were high (>90%) for most urinary phthalate metabolites, except for MEHP, which was detected in only 65% of samples. GM concentrations varied widely, with the highest observed for MEP (56.0 ng/mL) followed by MECPP (15.2 ng/mL) (Table 2). All analytes demonstrated positive correlations; MEHHP and MEOHP were the most strongly correlated (Spearman ρ = 0.98) (Supplemental Figure 2, https://links.lww.com/EE/A450).

**Table 2.** Urinary phthalate metabolite distribution, NHANES (2003–2018)

Urinary metabolite	Abbreviation	%>LOD	25th	GM (ng/mL)	75th
Individual metabolites					
Monoethyl phthalate	MEP	99.8%	18.60	56.0	156.80
Mono-n-butyl phthalate	MnBP	98.5%	5.80	11.32	25.18
Monoisobutyl phthalate	MiBP	98.0%	3.00	5.22	12.70
Monobenzyl phthalate	MBzP	97.6%	2.02	4.85	12.25
Mono(2-ethylhexyl) phthalate	MEHP	65.3%	0.60	1.59	3.20
Mono(2-ethyl-5-hydroxyhexyl) phthalate	MEHHP	99.4%	4.04	9.73	21.79
Mono(2-ethyl-5-oxyhexyl) phthalate	MEOHP	99.0%	2.60	6.07	13.30
Mono(2-ethyl-5-carboxypentyl) phthalate	MECCP	99.8%	6.60	15.15	32.30
Summary measures					
Di(2-ethylhexyl) phthalate metabolites (MEHP, MEHHP, MEOHP, MECPP)	∑*DEHP*	99.7%	0.03	0.11	0.16
Personal care product–associated phthalate metabolites (MnBP, MEP, MiBP)	∑*PCP*	100%	0.18	0.43	1.03
Butyl group phthalate metabolites (MnBP, MiBP, MBzP)	∑*Butyls*	99.7%	0.06	0.11	0.23
Plastic-associated phthalate metabolites (MEHP, MEHHP, MEOHP, MECPP, MBzP)	∑*Plastics*	99.8%	0.06	0.14	0.30

%>LOD = weighted percent of samples above the limit of detection; 25th = 25th percentile; GM = geometric mean; 75th = 75th percentile. ∑ = sum of group metabolites.

### Associations of food security with phthalate metabolite concentrations (249)

In creatinine-adjusted models, several phthalates, including MiBP, MBzP, and ∑*Butyls*, exhibited positive associations with decreasing food security (e.g., for MBzP, percent difference: marginal = 11.8%, low = 18.3%, very low = 41.0%, vs. high food security, Supplemental Table 1, https://links.lww.com/EE/A450). Associations varied across food security levels for the remaining analytes (e.g., for ∑*DEHP*, percent difference: marginal = –8.9%, low = –12.5%, very low = –9.0%, vs. high food secure, Supplemental Table 1, https://links.lww.com/EE/A450).

**Figure 1.**
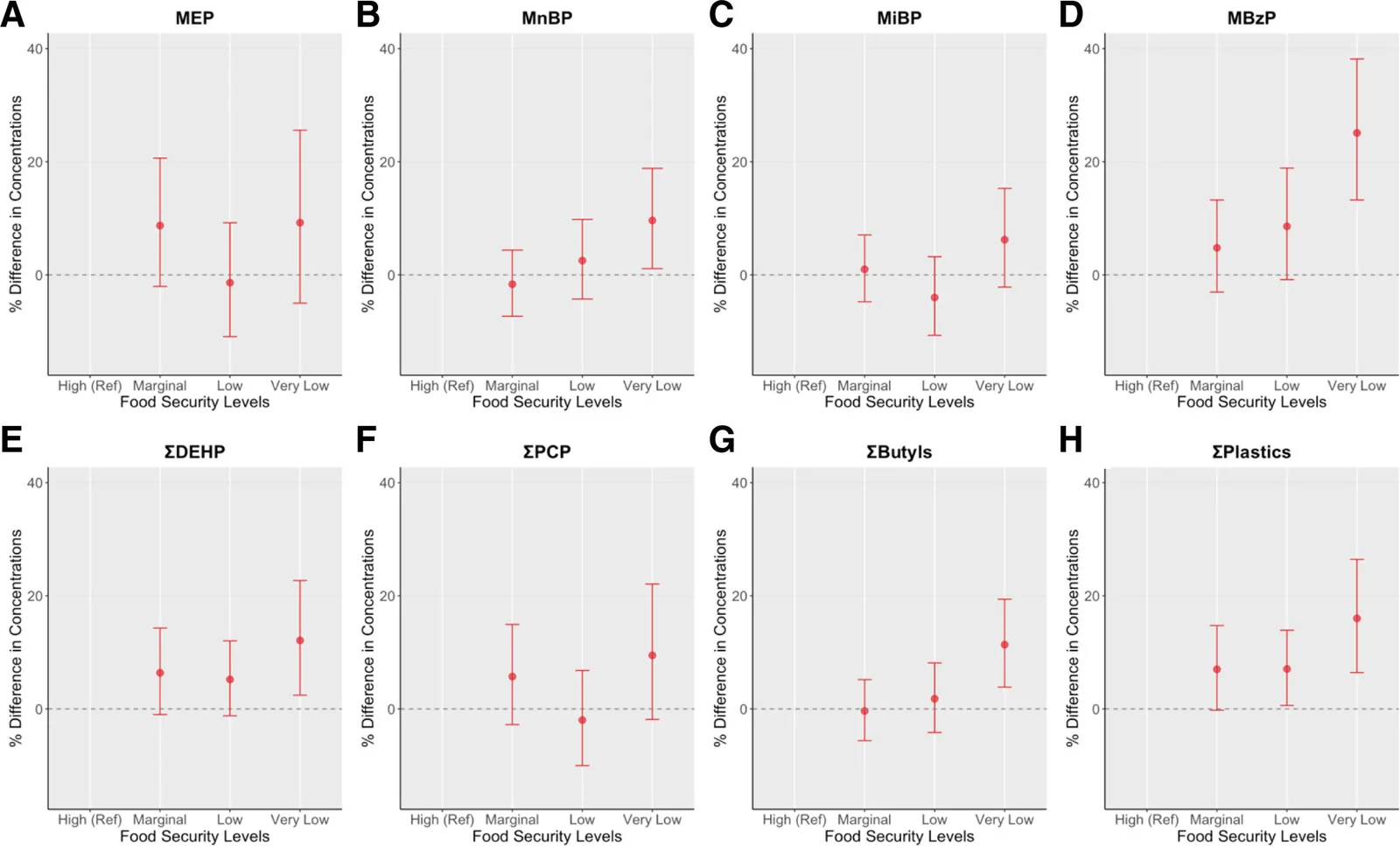
Adjusted linear regression: self-reported food security status and urinary phthalate metabolites. Adjusted percent differences (95% confidence intervals) in urinary phthalate metabolite concentrations across food security levels, estimated from multivariable linear regression models adjusted for age, sex, education, survey cycle, race, ethnicity, poverty:income ratio, and urinary creatinine. High food secure is the reference group.

In models adjusting for demographic covariates, several of these patterns persisted. Notably, individuals experiencing very low food security had significantly higher urinary concentrations of MBzP (25.1%; 95% CI: 13.2%, 38.2%) and MnBP (9.6%; 95% CI: 1.1%, 18.8%) compared with those with high food security (Figure 1, Supplemental Table 2, https://links.lww.com/EE/A450). In contrast, other individual analytes, including MiBP and MEP, demonstrated variable associations across food security levels, with individuals experiencing low, but not very low, food security having lower levels compared with those with high food security (MiBP: −4.0%, 95% CI: −10.7%, 3.2% and MEP: −1.4%, 95% CI: −10.9%, 9.2%), although these associations did not reach statistical significance.

With respect to the summary measures, individuals experiencing very low food security had higher concentrations of both ∑*DEHP* (12.1%; 95% CI: 2.4%, 22.7%), ∑*Butyls* (11.4%; 95% CI: 3.9%, 19.4%), and ∑*Plastics* (low: 7.1%; 95% CI: 0.6%, 13.9%; very low: 16.0%; 95% CI: 6.4%, 26.4%) compared with those with high food security (Figure 1, Supplemental Table 2, https://links.lww.com/EE/A450). No significant associations were observed for other individual analytes or summary measures (Table 3, Supplemental Table 2, https://links.lww.com/EE/A450).

### Effect modification by sex

**Figure 2.**
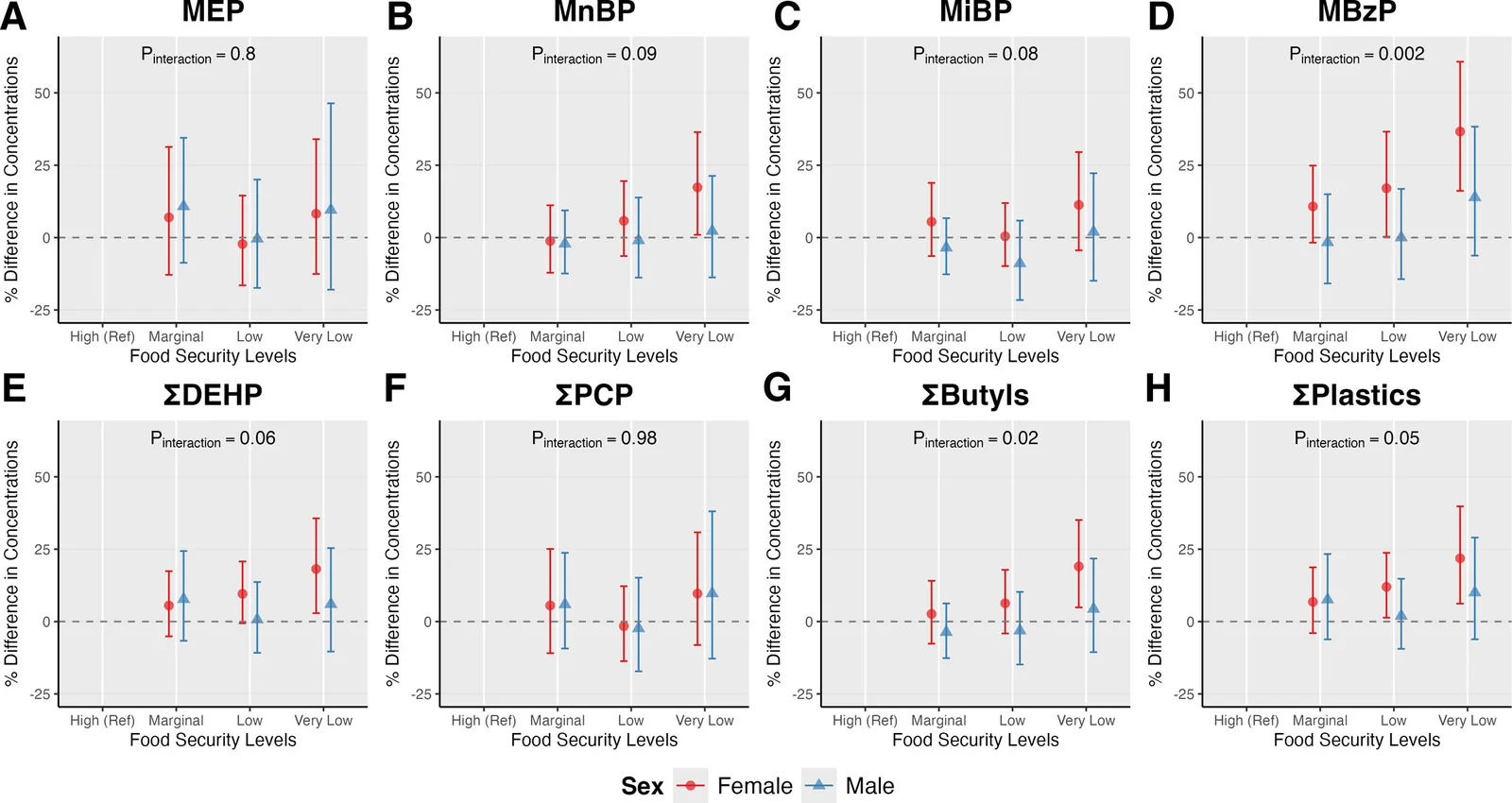
Strata-specific adjusted linear regression plot: self-reported food security status and urinary phthalate metabolites with sex interaction. Sex-stratified adjusted percent differences (95% confidence intervals) in urinary phthalate metabolite concentrations across food security levels, estimated from multivariable linear regression models adjusted for age, race/ethnicity, education, survey cycle, poverty:income ratio, and creatinine. High Food Secure is the reference group. *P*_interaction_ values are reported from food security × sex interaction models, where food security is modeled as a continuous variable.

In evaluations of effect measure modification by sex, models for MBzP (*P*_interaction_ = 0.002) and MiBP (*P*_interaction_ = 0.08) revealed significant differences in trend over decreasing food security levels. Within strata-specific predicted marginal means, higher percent differences for MBzP (e.g., very low food security: 36.6%; 95% CI: 16.2%, 60.8%, Figure 2, Supplemental Table 3, https://links.lww.com/EE/A450) were observed among females experiencing very low food security compared with females with high food security. In MiBP models, females with very low food security showed suggestively higher estimated concentrations (very low food security: 11.3%; 95% CI: −4.41%, 29.5%, Figure 2, Supplemental Table 3, https://links.lww.com/EE/A450), with values across categories varying in magnitude but remaining generally positive. Estimates for males did not reach statistical significance for either butyl analyte or for any other individual analytes.

∑DEHP (*P*_interaction_ = 0.06), ∑Butyls (*P*_interaction_ = 0.02), and ∑Plastics (*P*_interaction_ = 0.05) exhibited significant sex by food security interactions across trends in fully adjusted models. Across all summary measures except ∑PCPs, statistically significant differences were observed among females with very low food security compared with the reference group, with higher concentrations reported: ∑DEHP (very low food security: 18.2%; 95% CI: 2.9%, 35.7%), ∑Butyls (very low food security: 19.0%; 95% CI: 4.9%, 35.1%), and ∑Plastics (very low food security: 21.8%; 95% CI: 6.2%, 39.8%) (Figure 2, Supplemental Table 3, https://links.lww.com/EE/A450). Associations in males were weaker and more variable than those observed in females.

### Effect modification by race/ethnicity

**Figure 3.**
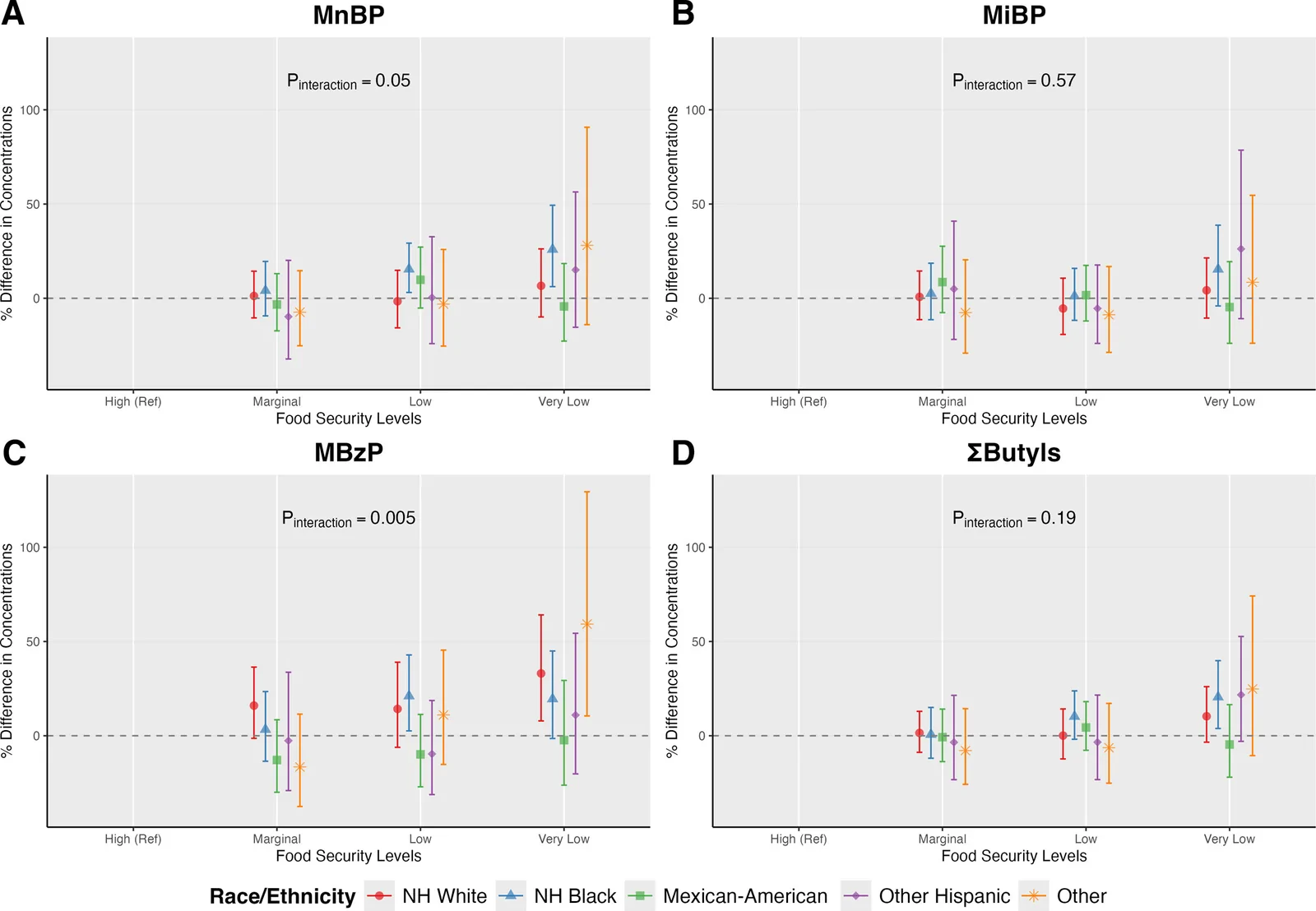
Strata-specific adjusted linear regression plot: self-reported food security status and urinary phthalate metabolites with race/ethnicity interaction. Race-stratified adjusted percent differences (95% confidence intervals) in urinary phthalate metabolite concentrations across food security levels, estimated from multivariable linear regression models adjusted for age, sex, education, survey cycle, poverty:income ratio, and creatinine. High Food Secure is the reference group. *P*_interaction_ values are reported from food security × race/ethnicity interaction models, where food security is modeled as a continuous variable. NH White: non-Hispanic White; NH Black: non-Hispanic Black.

Finally, we evaluated effect measure modification by race/ethnicity. A significant interaction was observed for MnBP (*P*_interaction_ = 0.05) and MBzP (*P*_interaction_ = 0.005), indicating that the association between food security and both metabolite levels differed by race/ethnicity. When evaluating race/ethnicity stratum-specific predicted marginal means, mixed results were seen overall (Supplemental Tables 4–11, https://links.lww.com/EE/A450). We observed higher levels of MnBP among non-Hispanic Black individuals with low food security (15.4%, 95% CI: 3.1, 29.2) and very low food security (25.9%, 95% CI: 6.2, 49.3) compared with those fully food secure (Figure 3, Supplemental Table 5, https://links.lww.com/EE/A450). Non-Hispanic White individuals (very low food security: 33.1%; 95% CI: 7.9%, 64.1%), non-Hispanic Black individuals (low food security: 21.1%; 95% CI: 2.6%, 42.9%), and individuals of Other races (very low food security: 59.3%; 95% CI: 10.5%, 129.5%) had significantly higher levels of MBzP compared with those with high food security (Figure 3, Supplemental Table 7, https://links.lww.com/EE/A450).

### Post hoc analyses

In post-hoc analyses, we explored supplement use and oral contraceptive (OC) use for potential confounding for select analytes including MBzP and MnBP. When comparing those who reported use of supplements vs. those who did not, we did not observe appreciable differences among the GMs for MnBP but did for MBzP among supplement users (Supplemental Table 12, https://links.lww.com/EE/A450). Survey-weighted chi-square analysis showed no significant association between food security status and supplement use (*P* = 0.16), and in fully adjusted models, supplement use was not associated with MBzP levels and likewise did not attenuate the results observed in our primary analysis (*data not shown*). With respect to OC use, mean log butyl phthalate levels were generally lower in participants not currently using OCs compared with OC users, particularly for MiBP (Supplemental Table 13, https://links.lww.com/EE/A450). However, the distribution of food security status did not significantly differ by OC use in survey-weighted chi-square analysis (*P* = 0.07) or fully adjusted models (*data not shown*).

Additionally, we explored the impact that food fasting status may have on the same select analytes. MBzP was associated with fasting status in unadjusted analysis (Supplemental Table 14, https://links.lww.com/EE/A450), but not in fully adjusted models that explored potential effect modification by including an interaction term between food security and fasting status (Supplemental Table 15, https://links.lww.com/EE/A450).

## Discussion

In this large, nationally representative cross-sectional analysis of NHANES data, we found that lower levels of food security were associated with higher urinary concentrations of butyl-associated phthalate metabolites, namely MBzP and MnBP, as well as ∑DEHP and ∑Plastics metabolites. These associations were independent of several other demographic markers, including educational status, age, race/ethnicity, as well as FIP ratio. In addition, certain population subgroups showed stronger associations between food security and urinary phthalate metabolite concentrations and their summary measures. Specifically, females showed stronger associations than males when assessing reduced food security and MiBP, MBzP, ∑DEHP, ∑Plastics, and ∑Butyls. Additional interaction by race/ethnicity was also observed. With over 19% of this representative sample of the US population reporting low or very low food security, these findings highlight the potentially higher exposure of certain phthalates, particularly those with known associations to food packaging and upstream food processing.^16^

Prior reports have not consistently identified strong associations between dietary intake and ∑Butyls phthalates as we reported.^42,43^ While BBzP is not typically associated with food exposures, the Food and Drug Administration previously listed it as an optional food packaging chemical.^44^ Diet has primarily been reported as a significant pathway for exposure to DEHP and alternatives.^17^ This is likely driven by the previously described “substitution effect”^2^ where food-insecure adult individuals tend to consume more added sugars, high-fat foods,^45^ fewer fruits,^46^ and vegetables.^47^ Food-insecure individuals often cycle between sufficient caloric intake from food of poor nutrient quality and hunger.^48^ Financial strain can increase the likelihood of purchasing processed and fast foods.^49^ This altered dietary behavior may increase exposure to metabolites of harmful environmental chemicals, including phthalates associated with food contact material, such as DEHP prevalent in processed,^14^ fast food,^15^ and packaged foods prepared outside the home, as opposed to minimally processed or fresh food options.^12^

Beyond findings from observational studies, a few intervention studies have investigated the association between whole food or vegetarian^42,50^ diet substitution and urinary phthalate metabolites. In an intervention by Rudel et al,^42^ 20 participants in five families underwent an intervention where processed foods (canned foods and foods with plastic packaging) were substituted with fresh ingredients. Urinary phthalate levels were assessed before, during, and after the intervention. The GM of urinary DEHP metabolite concentrations fell by over 50% among participants who received the intervention over 3 days, but no change was observed for other phthalate metabolites, including MnBP and MBzP, which coincides with our reports of no effect modification by fasting status for either analyte.^42^ These findings suggest that dietary sources of exposure, particularly for MBzP,^42^ may occur in preprocessing stages via contact with food processing equipment and materials,^42^ rather than through acute dietary intake alone. Although DBP is less frequently linked to food contact in US studies,^51^ the migration of both DBP and DEHP from plastic packaging of convenience foods in China^52^ suggests that dietary pathways cannot be entirely discounted.

We also considered whether other potential dietary sources, such as supplement use, may influence our findings specifically for the butyl-associated metabolites, as some low molecular weight phthalates may be used in supplement coatings.^53^ Post hoc analyses did not find supplement use to be linked to food security status. As such, the use of supplements was not likely to confound the association between food security and phthalate metabolite concentrations in this study population.

Alternatively, it is likely that our findings, particularly for the ∑Butyl-associated analytes, suggest that income is an important upstream factor linking food security with phthalate exposure. Tyrrell et al^54^ found negative associations between the poverty-to-income ratio and MBzP and MnBP among NHANES participants, though the role of diet was unclear. Still, in our analyses, associations between food security and select phthalates persisted after adjustment for the poverty-to-income ratio, indicating that food security may capture specific additional dimensions of socioeconomic position not fully reflected by income alone.

In our examination by sex, we found stronger associations for hypothesized diet-related metabolites, including ∑DEHP and ∑Plastic-associated, as well as MBzP and MnBP, which may reflect differential patterns in food purchasing,^55^ preparation,^55^ as well as possible physiological differences in phthalate metabolism.^56^ With respect to phthalates that may originate from ∑PCP-associated sources, previous studies have reported higher PCP use among females,^24^ with documented socioeconomic disparities,^57,58^ including by income status, in the types of products used^59^ and in associated phthalate metabolite concentrations.^45,47^ However, we did not observe differences in PCP-associated metabolites such as MEP, a primary predictor of PCP use. Alternatively, potential sex-based differences in time spent indoors and gendered roles also related to food preparation may contribute to higher exposure to dust from building materials, such as vinyl flooring, that contain butyl phthalates and are more commonly found in lower-quality housing.^16,22^

We also observed differences in trends across food security levels by race/ethnicity for MnBP and MBzP, similar to the overall population. Stratum-specific models revealed that MnBP levels were significantly higher among non-Hispanic Black participants experiencing reduced food security compared with food secure participants in the same group. This is consistent with findings demonstrating disparities in urinary phthalate metabolites among non-Hispanic Black individuals in other US populations^45,46^ and in NHANES,^49,60^ and may in part reflect built environment disparities, although the specific mechanism is unclear. With respect to MBzP, non-Hispanic White, non-Hispanic Black, and Other race individuals with reduced food security had higher MBzP levels than those with high food security, with stronger contrasts than in other racial and ethnic groups. As it relates to race/ethnicity, this may relate to variations in packaged food consumption given cultural preferences,^61^ food storage practices,^61,62^ alongside potential nondietary sources described above. Additional research is needed to understand the extent to which socioeconomic status dimensions, including housing characteristics,^16,43^ interact with food security to shape phthalate exposure.

### Limitations

The present study must be interpreted within the context of its cross-sectional design, which precludes establishing temporality between food security and urinary phthalate metabolite concentrations. Still, reverse causation is unlikely, as we are not aware of evidence that phthalate metabolism could affect food security status. While food security questionnaires were well validated,^29,30^ self-reported data may be subject to error in participant-recalled responses, potentially resulting in nondifferential misclassification that biases findings toward the null. Additionally, urinary phthalate metabolite concentrations were assessed via spot urine samples and may not account for temporal changes in exposure.^7^ Furthermore, given the short half-life of phthalates and high intraindividual variability inherent to spot urine samples,^16^ a single measurement may not adequately reflect usual exposure,^7^ potentially attenuating observed associations and warranting cautious interpretation of effect sizes. While we summarized several individual metabolites based on literature regarding common sources, biological mechanisms, and correlation structure, we cannot distinguish between food and other exposure sources, including personal care products. Additionally, although NHANES provides 24-hour dietary records, we did not utilize this data due to documented limitations in the accurate categorization of processed foods.^14,63,64^ Instead, our analysis focused on utilizing USDA food security status categories to directly address our central research question. Finally, we cannot rule out the possibility of residual confounding due to unmeasured factors, such as the use of certain household products, medications, or other environmental exposures,^65^ that were not captured in our data.

### Strengths

There are several strengths to this study. To our knowledge, this study is the first to utilize data from a large, nationally representative dataset spanning 18 years to estimate associations between household food security and eight individual urinary phthalate metabolite concentrations, as well as four summary measures. Household food security was ascertained using a widely validated questionnaire^29,30^ with a rigorous three-stage design and screeners to minimize response burden while prioritizing reliability.^29^ Urinary phthalate metabolite concentrations were analyzed using standardized CDC techniques, with most phthalate metabolites detected in over 90% of the sample population.^11^ We controlled for several potential confounders, including relevant sociodemographic factors, and explored associations within population subgroups (i.e., by sex and race/ethnicity).

## Conclusion

In summary, our analysis revealed that lower levels of household food security were associated with significantly elevated urinary concentrations of butyl phthalate metabolites, particularly MBzP and MnBP, as well as ∑DEHP and ∑Plastics metabolites. These associations were modified by sex and race/ethnicity, with females and certain racial and ethnic groups having stronger associations. These results highlight reduced food security as a potential source of phthalate exposure. As established endocrine disruptors that may pose significant health risks to exposed individuals,^66^ further research is needed to understand the extent to which food security shapes potential dietary-based chemical exposure and may interact with other sources of exposure that collectively contribute to disparities in phthalate exposure and related health outcomes.

## Supplementary Material


