# Supplementary material for: Household food security and urinary phthalate metabolite concentrations among US adults: Evidence from NHANES 2003–2018
Source: Environ Epidemiol. 2026 Sep 1;10(5):e512. doi: 10.1097/EE9.0000000000000512 (PMC13537720; doi:10.1097/EE9.0000000000000512)
Supplement: Supplementary file 1 [file ee9-10-e512-s001.pdf]

Supplemental Figure 1: Follow up graphic for NHANES sample population (2003-2018)

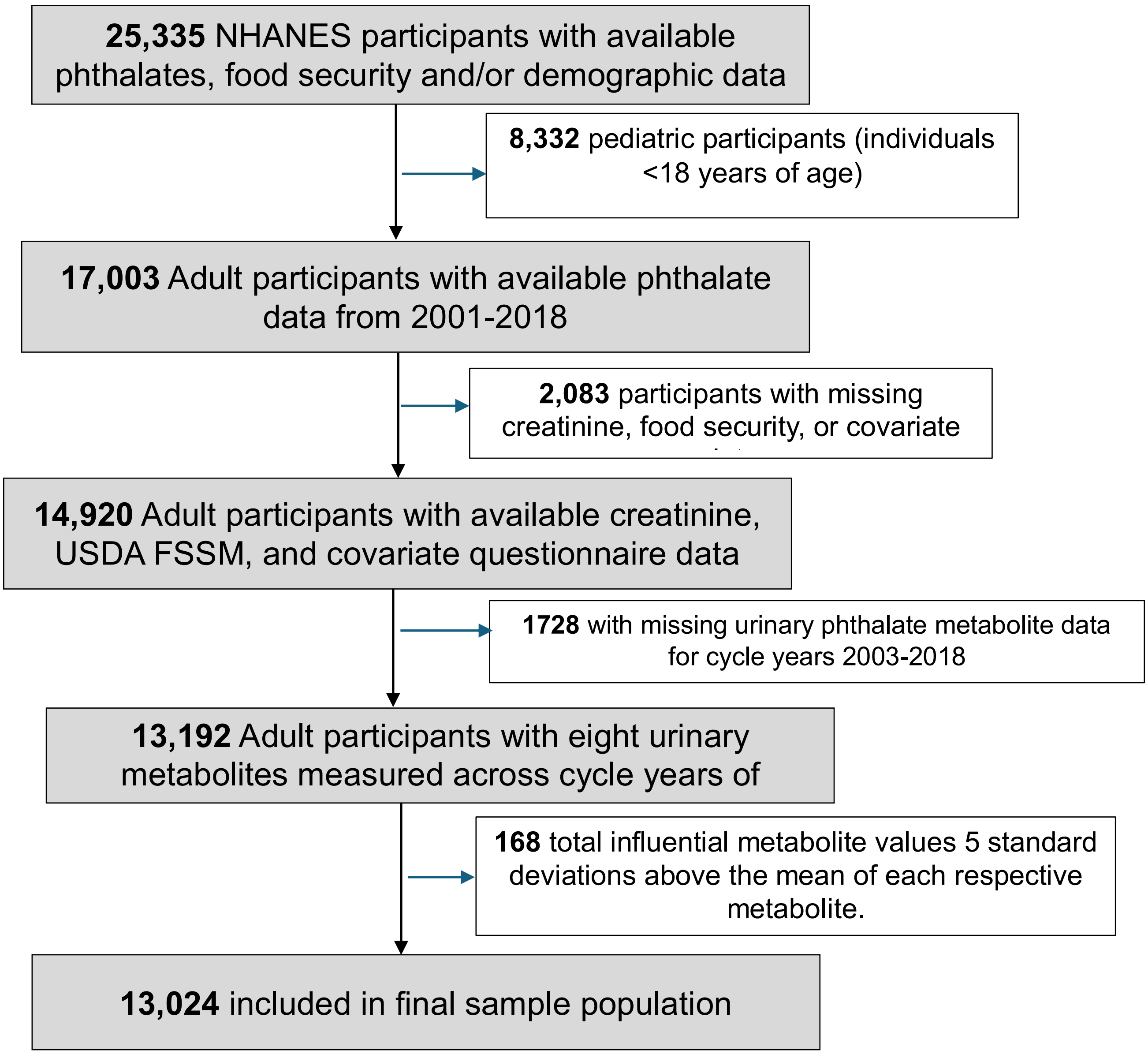

Adult participants from NHANES cycles 2003-2018 were included based on available demographic, food security, urinary phthalate and creatinine data. Exclusions were applied for missing exposure values, missing urinary creatinine and missing urinary phthalate metabolite concentrations as well as extreme outliers. Abbreviations: NHANES, National Health and Nutrition Examination Survey; USDA, United States Department of Agriculture; FSSM, Food Security Survey Module

Supplemental Figure 2: Spearman Correlation Matrix for Log Urinary Phthalate Metabolites in NHANES sample population (2003-2018)

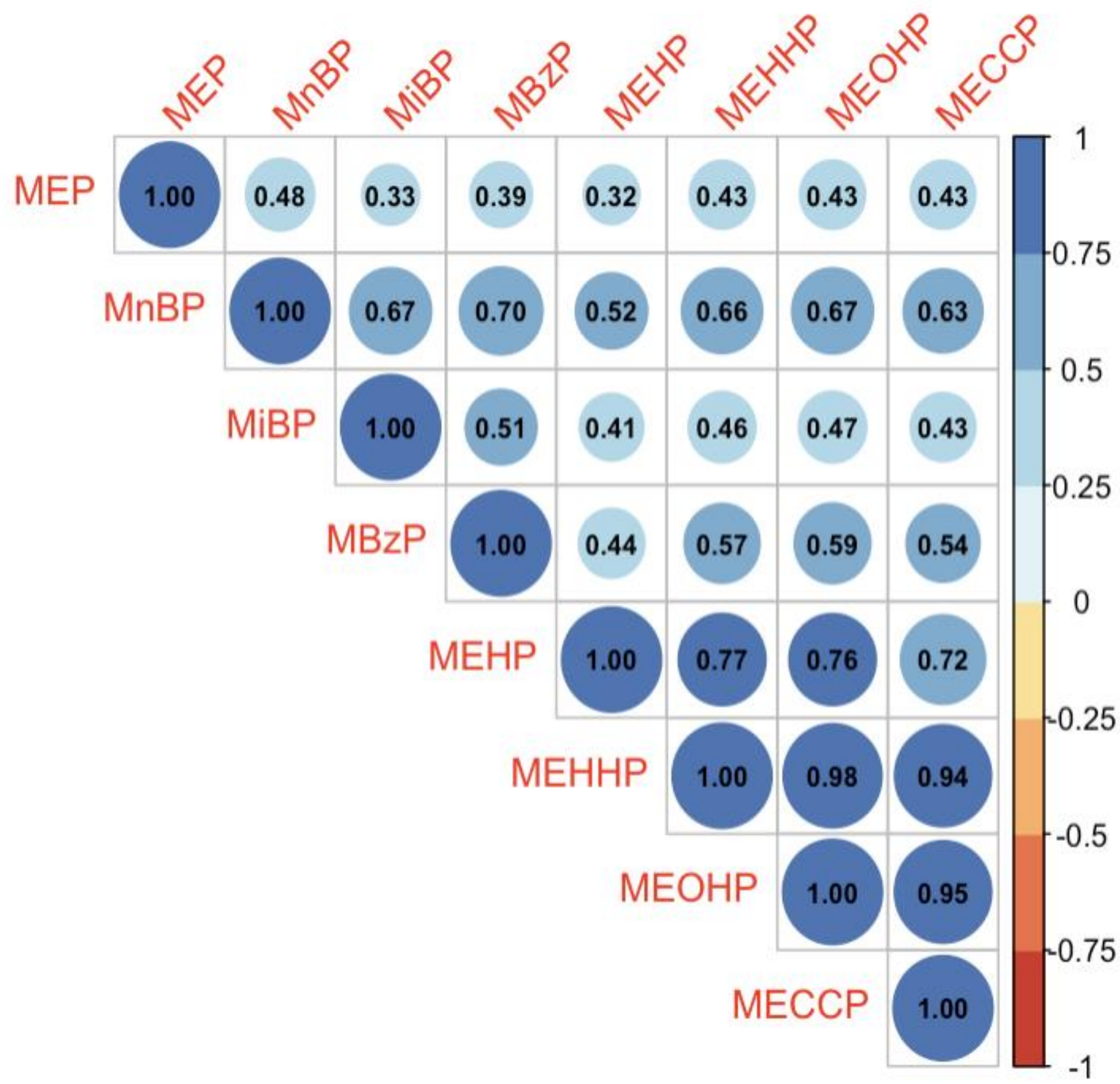

Spearman correlation coefficients are visualized, with larger and darker circles indicating stronger positive correlations between analytes. All correlations shown are positive.

Supplemental Table 1: Estimated creatinine-adjusted percent difference in urinary phthalate metabolite concentrations comparing lower vs. high food security among US adults, NHANES 2003–2018

Phthalate Metabolite	Food Security Level	% Difference	% Lower	% Higher
**MEP**	High (Ref)			
	Marginal	5.28	-6.86	19.00
	Low	-3.37	-13.66	8.14
	Very Low	1.94	-12.31	18.52
**MnBP**	High (Ref)			
	Marginal	-4.31	-11.12	3.02
	Low	2.42	-5.04	10.46
	Very Low	8.16	-2.08	19.48
**MiBP**	High (Ref)			
	Marginal	17.61	10.34	25.36
	Low	14.19	6.66	22.24
	Very Low	24.24	14.52	34.79
**MBzP**	High (Ref)			
	Marginal	11.84	2.69	21.82
	Low	18.33	7.41	30.37
	Very Low	41.01	27.23	56.27
**∑DEHP**	High (Ref)			
	Marginal	-8.89	-17.11	0.16
	Low	-12.50	-19.50	-4.91
	Very Low	-9.03	-17.67	0.52
**∑ PCP**	High (Ref)			
	Marginal	4.64	-5.60	15.99
	Low	-1.04	-10.07	8.91
	Very Low	6.85	-5.55	20.88
**∑Butyls**	High (Ref)			
	Marginal	4.14	-2.06	10.73
	Low	9.19	2.67	16.13
	Very Low	19.15	10.10	28.95
**∑Plastics**	High (Ref)			
	Marginal	-4.46	-12.76	4.63
	Low	-6.25	-13.36	1.44
	Very Low	0.34	-8.74	10.33

Values are creatinine adjusted percent differences in urinary phthalate metabolite concentrations (ng/mL) compared to the reference group (High Food Secure) based on multivariable linear regression models. % Lower and % Higher indicating the bounds of the 95% confidence interval.

Supplemental Table 2: Estimated adjusted percent difference in urinary phthalate metabolite concentrations comparing lower vs. high food security among US adults, NHANES 2003–2018

Phthalate Metabolite	Food Security Level	% Difference	95% CI	
MEP	High (Ref)			
	Marginal	8.71	-2.04	20.64
	Low	-1.38	-10.93	9.21
	Very Low	9.22	-5.01	25.57
MnBP	High (Ref)			
	Marginal	-1.65	-7.33	4.37
	Low	2.52	-4.28	9.80
	Very Low	9.61	1.10	18.84
MiBP	High (Ref)			
	Marginal	0.98	-4.75	7.05
	Low	-4.00	-10.70	3.20
	Very Low	6.20	-2.17	15.29
MBzP	High (Ref)			
	Marginal	4.78	-3.05	13.24
	Low	8.57	-0.84	18.88
	Very Low	25.09	13.24	38.18
∑DEHP	High (Ref)			
	Marginal	6.38	-0.99	14.30
	Low	5.20	-1.21	12.03
	Very Low	12.11	2.43	22.71
∑ PCP	High (Ref)			
	Marginal	5.71	-2.78	14.94
	Low	-1.97	-10.03	6.80
	Very Low	9.46	-1.86	22.08
∑Butyls	High (Ref)			
	Marginal	-0.36	-5.61	5.18
	Low	1.79	-4.18	8.13
	Very Low	11.36	3.85	19.40
∑Plastics	High (Ref)			
	Marginal	6.99	-0.24	14.75
	Low	7.05	0.60	13.91
	Very Low	16.00	6.42	26.44

Values are adjusted percent differences in urinary phthalate metabolite concentrations (ng/mL) compared to the reference group (High Food Secure) based on multivariable linear regression models adjusted for age, gender, education, cycle year, race/ethnicity, poverty: income ratio and creatinine. % Lower and % Higher indicating the bounds of the 95% confidence interval.

Supplemental Figure 3: Stratum specific estimated adjusted percent difference in urinary phthalate metabolites (ng/mL) comparing lower vs. high food security among US adults, NHANES 2003-2018 -- with race/ethnicity interaction

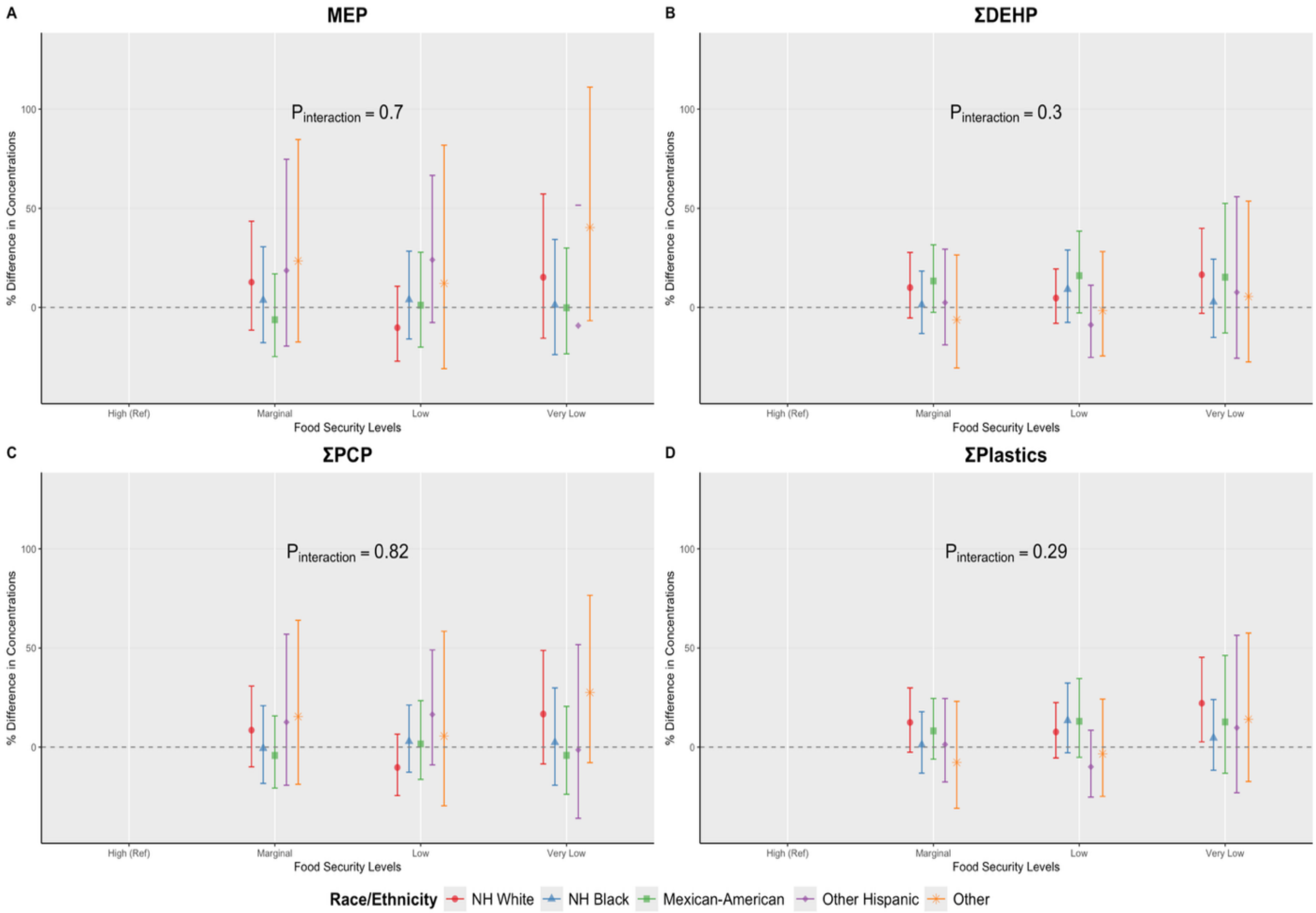

Race stratified adjusted percent differences (95% confidence intervals) in urinary phthalate metabolite concentrations across food security levels, estimated from multivariable linear regression models adjusted for age, sex, education, survey cycle, poverty:income ratio and creatinine. High Food Secure is the reference group. $P_{interaction}$ values are reported from food security*race/ethnicity interaction models, where food security is modeled as continuous variable. Abbreviations: NH White: Non-Hispanic White; NH Black: Non-Hispanic Black.

Supplemental Table 3: Stratum specific estimated adjusted percent difference in urinary phthalate metabolites (ng/mL) comparing lower vs. high food security among US adults, NHANES 2003-2018-- with sex interaction

Sex	Phthalate Metabolite	Food Security Level	% Difference (Interaction)	% Lower	% Higher
**Female**	**MEP**	High (Ref)			
		Marginal	6.97	-12.86	31.31
		Low	-2.22	-16.51	14.51
		Very Low	8.24	-12.57	34.01
**Male**		High (Ref)			
		Marginal	10.79	-8.71	34.46
		Low	-0.41	-17.39	20.05
		Very Low	9.56	-18.00	46.38
**Female**	**MnBP**	High (Ref)			
		Marginal	-1.19	-12.15	11.14
		Low	5.77	-6.39	19.51
		Very Low	17.36	0.97	36.43
**Male**		High (Ref)			
		Marginal	-2.12	-12.43	9.39
		Low	-0.96	-13.82	13.83
		Very Low	2.28	-13.77	21.32
**Female**	**MiBP**	High (Ref)			
		Marginal	5.49	-6.40	18.89
		Low	0.45	-9.86	11.93
		Very Low	11.28	-4.41	29.53
**Male**		High (Ref)			
		Marginal	-3.48	-12.69	6.70
		Low	-8.90	-21.59	5.86
		Very Low	1.98	-14.92	22.23
**Female**	**MBzP**	High (Ref)			
		Marginal	10.76	-1.75	24.86
		Low	17.03	0.27	36.58
		Very Low	36.64	16.15	60.76
**Male**		High (Ref)			
		Marginal	-1.64	-15.85	14.98
		Low	0.03	-14.35	16.83
		Very Low	13.90	-6.22	38.33
**Female**	**∑DEHP**	High (Ref)			
		Marginal	5.55	-5.12	17.41
		Low	9.58	-0.58	20.76
		Very Low	18.15	2.89	35.67

**Male**		High (Ref)			
		Marginal	7.75	-6.64	24.36
		Low	0.69	-10.8	13.66
		Very Low	6.02	-10.36	25.39
**Female**	**∑ PCP**	High (Ref)			
		Marginal	5.55	-10.94	25.10
		Low	-1.58	-13.68	12.22
		Very Low	9.65	-8.10	30.82
**Male**		High (Ref)			
		Marginal	5.94	-9.31	23.76
		Low	-2.35	-17.19	15.14
		Very Low	9.72	-12.81	38.08
**Female**	**∑Butyls**	High (Ref)			
		Marginal	2.64	-7.65	14.08
		Low	6.31	-4.12	17.87
		Very Low	19.04	4.89	35.09
**Male**		High (Ref)			
		Marginal	-3.65	-12.64	6.27
		Low	-3.11	-14.84	10.25
		Very Low	4.36	-10.56	21.76
**Female**	**∑Plastics**	High (Ref)			
		Marginal	6.78	-3.99	18.74
		Low	11.98	1.34	23.75
		Very Low	21.84	6.19	39.80
**Male**		High (Ref)			
		Marginal	7.59	-6.14	23.33
		Low	1.95	-9.43	14.77
		Very Low	10.05	-6.13	29.02

Values represent percent differences for Food Security Level × Sex interaction terms, showing strata-specific estimates. Within each sex group (female, male) estimates indicate the percent difference for each food security level compared with the reference. Estimates are derived from multivariable linear regression models adjusted for age, race/ethnicity, education, cycle year, poverty:income ratio and creatinine. Abbreviations: NH White: Non-Hispanic White; NH Black: Non-Hispanic Black. % Lower and % Higher indicating the bounds of the 95% confidence interval.

Supplemental Table 4: Stratum specific estimated adjusted percent difference in urinary phthalate metabolites (ng/mL) comparing lower vs. high food security among US adults, NHANES 2003-2018-- with race/ethnicity interaction

Race/ethnicity Category	Model	Food Security Contrast	% Difference	% Lower	% Higher
NH White	MEP	High (Ref)			
		Marginal	12.73	-11.42	43.47
		Low	-10.20	-27.11	10.65
		Very Low	15.24	-15.50	57.16
NH Black		High (Ref)			
		Marginal	3.64	-17.77	30.63
		Low	3.89	-15.92	28.36
		Very Low	1.22	-23.72	34.30
Mexican-American		High (Ref)			
		Marginal	-6.20	-24.77	16.94
		Low	1.13	-20.00	27.85
		Very Low	-0.18	-23.33	29.95
Other Hispanic		High (Ref)			
		Marginal	18.61	-19.48	74.70
		Low	24.01	-7.65	66.53
		Very Low	-9.17	-45.57	51.57
Other		High (Ref)			
		Marginal	23.48	-17.43	84.64
		Low	12.13	-30.85	81.82
		Very Low	40.36	-6.66	111.07

Values represent percent differences for Food Security Level × Race/ethnicity interaction terms, showing strata-specific estimates. Within each race/ethnicity group (NH White, NH Black, Mexican-American, Other Hispanic, Other) estimates indicate the percent difference for each food security level compared with the reference. Estimates are derived from multivariable linear regression models adjusted for age, sex, education, cycle year, poverty:income ratio and creatinine. Abbreviations: NH White: Non-Hispanic White; NH Black: Non-Hispanic Black% Lower and % Higher indicating the bounds of the 95% confidence interval.

Supplemental Table 5: Stratum specific estimated adjusted percent difference in urinary phthalate metabolites (ng/mL) comparing lower vs. high food security among US adults, NHANES 2003-2018-- with race/ethnicity interaction

Race/ethnicity Category	Model	Food Security Contrast	% Difference	% Lower	% Higher
NH White	MnBP	High (Ref)			
		Marginal	1.23	-10.41	14.37
		Low	-1.60	-15.69	14.85
		Very Low	6.62	-9.92	26.21
NH Black		High (Ref)			
		Marginal	4.11	-9.33	19.55
		Low	15.44	3.09	29.26
		Very Low	25.93	6.18	49.34
Mexican-American		High (Ref)			
		Marginal	-3.27	-17.26	13.07
		Low	9.81	-5.18	27.18
		Very Low	-4.32	-22.70	18.44
Other Hispanic		High (Ref)			
		Marginal	-9.76	-32.21	20.13
		Low	0.37	-24.06	32.65
		Very Low	15.04	-15.39	56.42
Other		High (Ref)			
		Marginal	-7.39	-25.18	14.63
		Low	-3.07	-25.36	25.88
		Very Low	28.09	-14.00	90.77

Values represent percent differences for Food Security Level × Race/ethnicity interaction terms, showing strata-specific estimates. Within each race/ethnicity group (NH White, NH Black, Mexican-American, Other Hispanic, Other) estimates indicate the percent difference for each food security level compared with the reference. Estimates are derived from multivariable linear regression models adjusted for age, sex, education, cycle year, poverty:income ratio and creatinine. Abbreviations: NH White: Non-Hispanic White; NH Black: Non-Hispanic Black% Lower and % Higher indicating the bounds of the 95% confidence interval.

Supplemental Table 6: Stratum specific estimated adjusted percent difference in urinary phthalate metabolites (ng/mL) comparing lower vs. high food security among US adults, NHANES 2003-2018-- with race/ethnicity interaction

Race/ethnicity Category	Model	Food Security Contrast	% Difference	% Lower	% Higher
NH White	MiBP	High (Ref)			
		Marginal	0.74	-11.33	14.45
		Low	-5.43	-19.18	10.67
		Very Low	4.22	-10.52	21.39
NH Black		High (Ref)			
		Marginal	2.53	-11.37	18.60
		Low	1.16	-11.70	15.88
		Very Low	15.39	-4.06	38.79
Mexican-American		High (Ref)			
		Marginal	8.59	-7.61	27.63
		Low	1.64	-12.05	17.46
		Very Low	-4.67	-23.91	19.44
Other Hispanic		High (Ref)			
		Marginal	4.96	-21.83	40.93
		Low	-5.40	-23.93	17.66
		Very Low	26.21	-10.81	78.60
Other		High (Ref)			
		Marginal	-7.63	-29.13	20.40
		Low	-8.72	-28.70	16.85
		Very Low	8.49	-23.86	54.60

Values represent percent differences for Food Security Level × Race/ethnicity interaction terms, showing strata-specific estimates. Within each race/ethnicity group (NH White, NH Black, Mexican-American, Other Hispanic, Other) estimates indicate the percent difference for each food security level compared with the reference. Estimates are derived from multivariable linear regression models adjusted for age, sex, education, cycle year, poverty:income ratio and creatinine. Abbreviations: NH White: Non-Hispanic White; NH Black: Non-Hispanic Black. % Lower and % Higher indicating the bounds of the 95% confidence interval.

Supplemental Table 7: Stratum specific estimated adjusted percent difference in urinary phthalate metabolites (ng/mL) comparing lower vs. high food security among US adults, NHANES 2003-2018-- with race/ethnicity interaction

Race/ethnicity Category	Model	Food Security Contrast	% Difference	% Lower	% Higher
NH White	MBzP	High (Ref)			
		Marginal	16.00	-1.36	36.42
		Low	14.23	-6.13	39.01
		Very Low	33.06	7.88	64.11
NH Black		High (Ref)			
		Marginal	3.32	-13.53	23.45
		Low	21.08	2.60	42.88
		Very Low	19.53	-1.45	44.97
Mexican-American		High (Ref)			
		Marginal	-12.85	-29.98	8.47
		Low	-9.90	-27.08	11.33
		Very Low	-2.32	-26.22	29.34
Other Hispanic		High (Ref)			
		Marginal	-2.62	-29.08	33.70
		Low	-9.63	-31.20	18.70
		Very Low	10.97	-20.24	54.38
Other		High (Ref)			
		Marginal	-16.54	-37.52	11.48
		Low	11.05	-15.22	45.46
		Very Low	59.25	10.53	129.45

Values represent percent differences for Food Security Level × Race/ethnicity interaction terms, showing strata-specific estimates. Within each race/ethnicity group (NH White, NH Black, Mexican-American, Other Hispanic, Other) estimates indicate the percent difference for each food security level compared with the reference. Estimates are derived from multivariable linear regression models adjusted for age, sex, education, cycle year, poverty:income ratio and creatinine. Abbreviations: NH White: Non-Hispanic White; NH Black: Non-Hispanic Black. % Lower and % Higher indicating the bounds of the 95% confidence interval.

Supplemental Table 8: Stratum specific estimated adjusted percent difference in urinary phthalate metabolites (ng/mL) comparing lower vs. high food security among US adults, NHANES 2003-2018-- with race/ethnicity interaction

Race/ethnicity Category	Model	Food Security Contrast	% Difference	% Lower	% Higher
NH White	∑DEHP	High (Ref)			
		Marginal	10.00	-5.27	27.73
		Low	4.79	-8.02	19.39
		Very Low	16.52	-2.95	39.89
NH Black		High (Ref)			
		Marginal	1.39	-13.12	18.33
		Low	9.17	-7.58	28.94
		Very Low	2.77	-15.08	24.36
Mexican-American		High (Ref)			
		Marginal	13.31	-2.45	31.62
		Low	16.04	-2.75	38.46
		Very Low	15.28	-12.85	52.49
Other Hispanic		High (Ref)			
		Marginal	2.45	-18.86	29.35
		Low	-8.80	-25.18	11.16
		Very Low	7.64	-25.65	55.83
Other		High (Ref)			
		Marginal	-6.25	-30.51	26.47
		Low	-1.56	-24.39	28.15
		Very Low	5.54	-27.48	53.60

Values represent percent differences for Food Security Level × Race/ethnicity interaction terms, showing strata-specific estimates. Within each race/ethnicity group (NH White, NH Black, Mexican-American, Other Hispanic, Other) estimates indicate the percent difference for each food security level compared with the reference. Estimates are derived from multivariable linear regression models adjusted for age, sex, education, cycle year, poverty:income ratio and creatinine. Abbreviations: NH White: Non-Hispanic White; NH Black: Non-Hispanic Black. % Lower and % Higher indicating the bounds of the 95% confidence interval.

Supplemental Table 9: Stratum specific estimated adjusted percent difference in urinary phthalate metabolites (ng/mL) comparing lower vs. high food security among US adults, NHANES 2003-2018-- with race/ethnicity interaction

Race/ethnicity Category	Model	Food Security Contrast	% Difference	% Lower	% Higher
**NH White**	**∑PCP**	High (Ref)			
		Marginal	8.55	-9.93	30.81
		Low	-10.25	-24.39	6.54
		Very Low	16.69	-8.48	48.78
**NH Black**		High (Ref)			
		Marginal	-0.60	-18.28	20.92
		Low	2.91	-12.63	21.22
		Very Low	2.41	-19.19	29.79
**Mexican-American**		High (Ref)			
		Marginal	-4.15	-20.63	15.76
		Low	1.65	-16.30	23.46
		Very Low	-4.11	-23.71	20.51
**Other Hispanic**		High (Ref)			
		Marginal	12.60	-19.20	56.90
		Low	16.47	-8.96	49.00
		Very Low	-1.39	-35.90	51.72
**Other**		High (Ref)			
		Marginal	15.44	-18.72	63.96
		Low	5.63	-29.57	58.42
		Very Low	27.55	-7.84	76.53

Values represent percent differences for Food Security Level × Race/ethnicity interaction terms, showing strata-specific estimates. Within each race/ethnicity group (NH White, NH Black, Mexican-American, Other Hispanic, Other) estimates indicate the percent difference for each food security level compared with the reference. Estimates are derived from multivariable linear regression models adjusted for age, sex, education, cycle year, poverty:income ratio and creatinine. Abbreviations: NH White: Non-Hispanic White; NH Black: Non-Hispanic Black. % Lower and % Higher indicating the bounds of the 95% confidence interval.

Supplemental Table 10: Stratum specific estimated adjusted percent difference in urinary phthalate metabolites (ng/mL) comparing lower vs. high food security among US adults, NHANES 2003-2018-- with race/ethnicity interaction

Race/ethnicity Category	Model	Food Security Contrast	% Difference	% Lower	% Higher
NH White	∑Butyls	High (Ref)			
		Marginal	1.48	-8.81	12.95
		Low	0.09	-12.30	14.24
		Very Low	10.34	-3.41	26.05
NH Black		High (Ref)			
		Marginal	0.66	-11.92	15.03
		Low	10.23	-1.86	23.82
		Very Low	20.51	3.86	39.84
Mexican-American		High (Ref)			
		Marginal	-0.75	-13.71	14.15
		Low	4.40	-7.71	18.10
		Very Low	-4.66	-21.99	16.51
Other Hispanic		High (Ref)			
		Marginal	-3.48	-23.29	21.44
		Low	-3.40	-23.26	21.59
		Very Low	21.71	-2.97	52.68
Other		High (Ref)			
		Marginal	-7.86	-25.78	14.38
		Low	-6.35	-25.15	17.17
		Very Low	24.83	-10.53	74.17

Values represent percent differences for Food Security Level × Race/ethnicity interaction terms, showing strata-specific estimates. Within each race/ethnicity group (NH White, NH Black, Mexican-American, Other Hispanic, Other) estimates indicate the percent difference for each food security level compared with the reference. Estimates are derived from multivariable linear regression models adjusted for age, sex, education, cycle year, poverty:income ratio and creatinine. Abbreviations: NH White: Non-Hispanic White; NH Black: Non-Hispanic Black. % Lower and % Higher indicating the bounds of the 95% confidence interval.

Supplemental Table 11: Stratum specific estimated adjusted percent difference in urinary phthalate metabolites (ng/mL) comparing lower vs. high food security among US adults, NHANES 2003-2018-- with race/ethnicity interaction

Race/ethnicity Category	Model	Food Security Contrast	% Difference	% Lower	% Higher
NH White	∑Plastics	High (Ref)			
		Marginal	12.46	-2.56	29.81
		Low	7.66	-5.40	22.51
		Very Low	22.12	2.66	45.29
NH Black		High (Ref)			
		Marginal	1.21	-13.09	17.85
		Low	13.40	-2.83	32.34
		Very Low	4.68	-11.63	24.00
Mexican-American		High (Ref)			
		Marginal	8.18	-6.04	24.55
		Low	13.02	-5.10	34.59
		Very Low	12.69	-13.14	46.22
Other Hispanic		High (Ref)			
		Marginal	1.32	-17.56	24.52
		Low	-9.92	-25.23	8.51
		Very Low	9.75	-22.97	56.37
Other		High (Ref)			
		Marginal	-7.71	-30.81	23.10
		Low	-3.36	-24.82	24.23
		Very Low	14.07	-17.36	57.46

Values represent percent differences for Food Security Level × Race/ethnicity interaction terms, showing strata-specific estimates. Within each race/ethnicity group (NH White, NH Black, Mexican-American, Other Hispanic, Other) estimates indicate the percent difference for each food security level compared with the reference. Estimates are derived from multivariable linear regression models adjusted for age, sex, education, cycle year, poverty:income ratio and creatinine. Abbreviations: NH White: Non-Hispanic White; NH Black: Non-Hispanic Black. % Lower and % Higher indicating the bounds of the 95% confidence interval.

Supplemental Table 12: Differences in log-transformed urinary phthalate metabolites concentrations (ng/mL) by supplement use: survey-weighted t-test estimates

Metabolite	Difference in Mean (log)	95% CI	P-value
**MBzP**	0.34	[0.07, 0.63]	0.02
**MiBP**	0.03	[-0.22, 0.16]	0.73
**MnBP**	0.06	[-0.28, 0.17]	0.63

Values represent mean differences in log-transformed urinary phthalate metabolite concentrations (ng/mL) between supplement users based on survey-weighted t-test estimates. 95% CI = 95% confidence interval

Supplemental Table 13: Differences in log-transformed urinary phthalate metabolites (ng/mL) concentrations by oral contraceptive (OC) use: survey-weighted t-test estimates

Metabolite	Difference in Mean (log)	95% CI	P-value
**MBzP**	-0.17	[-0.08, 0.40]	0.19
**MiBP**	-0.25	[0.05, 0.46]	0.01
**MnBP**	-0.22	[-0.01, 0.45]	0.05

Values represent mean differences in log-transformed urinary phthalate metabolite concentrations (ng/mL) between oral contraceptive users based on survey-weighted t-test estimates. 95% CI = 95% confidence interval

Supplemental Table 14: Creatinine-adjusted % difference in urinary phthalate metabolites concentrations (ng/mL) comparing fasting (vs non-fasting) adults

Outcome	% Difference	% Lower	% Higher
**MEP**	0.53%	-0.09%	1.15%
**MBzP**	0.42%	-0.03%	0.88%
**MiBP**	-0.90%	-1.30%	-0.50%
**MnBP**	-0.07%	-0.53%	0.39%
**∑DEHP**	-0.73%	-1.18%	-0.28%
**∑PCP**	0.29%	-0.23%	0.82%
**∑Butyls**	-0.13%	-0.50%	0.25%
**∑Plastics**	-0.51%	-0.93%	-0.08%

Values represent creatinine-adjusted differences in urinary phthalate metabolite concentrations (ng/mL) comparing fasting (vs non-fasting) adults. % Lower and % Higher indicating the bounds of the 95% confidence interval.

Supplemental Table 15. Adjusted % difference in urinary phthalate metabolites concentrations (ng/mL) comparing fasting (vs non-fasting) adults

Outcome	% Difference	% Lower	% Higher
**MEP**	-0.19%	-0.75%	0.36%
**MnBP**	-0.14%	-0.56%	0.29%
**MiBP**	-0.04%	-0.43%	0.35%
**MBzP**	0.08%	-0.44%	0.59%
**∑DEHP**	-0.31%	-0.72%	0.11%
**∑PCP**	-0.20%	-0.66%	0.27%
**∑Butyls**	-0.08%	-0.45%	0.30%
**∑Plastics**	-0.15%	-0.57%	0.26%

Values represent creatinine-adjusted differences in urinary phthalate metabolite concentrations (ng/mL) comparing fasting (vs non-fasting) adults. % Lower and % Higher indicating the bounds of the 95% confidence interval. Estimates are derived from multivariable linear regression models adjusted for age, sex, education, cycle year, poverty:income ratio and creatinine with fasting status interaction term.
